# Selenium(IV) Polybromide Complexes: Structural Diversity Driven by Halogen and Chalcogen Bonding

**DOI:** 10.3390/molecules27165355

**Published:** 2022-08-22

**Authors:** Nikita A. Korobeynikov, Andrey N. Usoltsev, Alexander S. Novikov, Pavel A. Abramov, Maxim N. Sokolov, Sergey A. Adonin

**Affiliations:** 1 Nikolaev Institute of Inorganic Chemistry SB RAS, 630090 Novosibirsk, Russia; 2 Institute of Chemistry, Saint Petersburg State University, 199034 Saint Petersburg, Russia; 3 Joint Research Institute of Chemistry, Faculty of Physics, Mathematics and Natural Sciences, People’s Friendship University of Russia (RUDN University), 117198 Moscow, Russia

**Keywords:** polyhalogens, halogen bonding, non-covalent interactions, selenium, chalcogen bonding

## Abstract

Reactions between bromoselenate(IV)-containing solutions, dibromine and salts of pyridinium-family organic cations resulted in structurally diverse, bromine-rich polybromine-bromoselenates(IV): (4-MePyH)_5_[Se_2_Br_9_][SeBr_6_](Br_3_)_2_ (**1**), (2-MePyH)_2_{[SeBr_6_](Br_2_)} (**2**), (PyH)_2_{[SeBr_5_]Br(Br_2_)_2_} (**3**), (1-MePy)_2_{[SeBr_6_](Br_2_)} (**4**). The compounds feature halogen and (in the case of **3**) chalcogen bonding in solid state, resulting in formation of supramolecular architectures of different dimensionality. DFT calculations allowed estimation of the energies of non-covalent interactions in **1**–**4**; additionally, characterization by Raman spectroscopy was performed.

## 1. Introduction

Ability of homo- or heteroleptic halide complexes to form associates with di- or polyhalogens was discovered far before the formulation of the modern concepts of supramolecular chemistry (in particular, halogen bonding (XB), which stands beyond this phenomenon). Among the earliest examples, we can highlight the work by Petzold: [1] treating bromoantimonate(III) solutions by dibromine in presence of salts of different organic cations (mostly of pyridinium family), he isolated dark crystalline solids with unusually high bromine content (such as “SbBr_9_”) and confirmed the presence of polybromide species in these matters via redox titration. Later, the progress in XRD techniques allowed the group of researchers from the University of Iowa to re-discover these compounds: following from the structural data, bromoantimonate anions were accompanied by dibromine [2] or tribromide [3,4] units. The authors noticed unusually short Br···Br distances in these structures. At the same time, similar observations were made by von Schnering et al. [5] while working with tungsten bromide clusters. Since then, there appeared dozens of examples of crystal structures featuring halide ligand···polyhalogen interactions, but this information remained “unsorted” until 2018, when we presented a review on this topic [6]. The appearance of this summary followed the interest on polyhalogen-halometalates, which increased over the last decade. The works by the group of Feldmann [7,8,9,10] and Shevelkov [11,12,13], as well as our team [14,15,16], clearly revealed that ability to form supramolecular associates with di- or polyhalogens in solid state is rather common for halometalates of d- and, especially, *p*-block elements. Diversity of such compounds is especially rich in the case of iodine- and bromine-rich substances, but corresponding complexes can be obtained also with dichlorine (after the very first work by Weiss et al. [17], we recently provided additional examples of dichlorine-chlorometalates, as well as theoretical insights into the nature of Cl_2_ bonding in these substances [18,19]).

Currently, polyhalogen-halometalates are known for many elements, including metalloids (Te). In the course of our work, we were curious whether this chemistry can be expanded towards the elements of the periodic table with even less metallic properties. For selenium, there are two examples [20,21] of dibromine-bromoselenates(IV) (both based on [Se_2_Br_10_]^2-^) as well as one dichlorine-containing complex reported by us very recently [22]. We decided to check whether 1) new representatives of the family of dibromine-bromoselenates can be prepared via the methods we utilized for Te(IV) derivatives and 2) if this idea works, how structurally similar or different from those of Te(IV) such complexes will be.

Hereby, we present four dibromine-bromoselenates(IV): (4-MePyH)_5_[Se_2_Br_9_][SeBr_6_](Br_3_)_2_ (**1**), (2-MePyH)_2_{[SeBr_6_](Br_2_)} (**2**), (PyH)_2_{[SeBr_5_]Br(Br_2_)_2_} (**3**), (1-MePy)_2_{[SeBr_6_](Br_2_)} (**4**).

## 2. Materials and Methods

All reagents were obtained from commercial sources and used as purchased. Solvents were purified according to the standard procedures. 1-methylpyridinium iodide (1-MePyI) was prepared by reaction of pyridine and methyl iodide (1:1.05) with nearly quantitative yield. In all cases, concentrated aqueous HBr was used. Caution: the work with dibromine and its solutions, as well as with concentrated HBr, requires obligatory use of fume hood and adequate eye and skin protection (goggles and gloves). Selenium dioxide is toxic; compounds **1**–**4** must be treated as toxic as well.

### 2.1. Synthesis of **1**

In total, 111 mg (1 mmol) of SeO_2_ and 195 μL (2 mmol) of 4-MePy were dissolved in 6 mL of HBr at 70 °C (30 min). After that, 1.59 g of Br_2_ (10 mmol, 0.5 mL, 10× excess to Se) were added dropwise, and the mixture was slowly cooled to r.t. Within several hours, reddish-black crystals of **1** were formed; yield was 69%.

### 2.2. Synthesis of **2**

In total, 55 mg (0.5 mmol) of SeO_2_ and 99 μL (1 mmol) of 2-MePy were dissolved in 6 mL of HBr at 70 °C (30 min). After that, 0.79 g of Br_2_ (5 mmol, 0.25 mL, 10× excess to Se) were added dropwise, and the mixture was slowly cooled to r.t. Within several hours, there form reddish-black crystals of **2**, decomposing rapidly while kept outside the dibromine-containing mother liquor (see Discussion).

### 2.3. Synthesis of **3**

The procedure was the same as for **2**, using pyridine (81 μL, 1 mmol) instead of 2-MePy. Reddish-black crystals of **3**, decomposing rapidly while kept outside the dibromine-containing mother liquor (see Discussion), form within several hours after cooling the reaction mixture to r.t.

### 2.4. Synthesis of **4**

In total, 111 mg (0.5 mmol) of 1-MePyI were dissolved in 4 mL of water. After addition of 97 mg (0.58 mmol) of AgNO_3_, the mixture was stirred for 15 min. AgI was filtered off; to mother liquor, 2 mL of HBr was added and AgBr was filtered off. Solution was evaporated to dryness, and residue was dissolved in 3 mL of HBr, followed by the addition of 28 mg (0.25 mmol) of SeO_2_. The mixture was heated to 70 °C; then, 0.39 g (2.5 mmol, 0.13 mL) of Br_2_ was added dropwise and the mixture was slowly cooled to r.t., resulting in the formation of dark cherry-red crystals of **4** within several hours. Yield was 79%. For C_12_H_16_Br_8_N_2_Se calcd, %: C, 16.01; H, 1.79; N, 3.11, found, %: C, 16.14, H, 1.90; N, 3.21.

### 2.5. X-ray Diffractometry

Crystallographic data and refinement details for **1**–**4** are given in Table S1 (Supplementary Materials). The diffraction data were collected on a Bruker D8 Venture diffractometer with a CMOS PHOTON III detector and IµS 3.0 source (Mo Kα radiation, λ = 0.71073 Å) at 150 K. The φ- and ω-scan techniques were employed. Absorption correction was applied by SADABS (Bruker Apex3 software suite: Apex3, SADABS-2016/2 and SAINT, version 2018.7-2; Bruker AXS Inc.: Madison, WI, USA, 2017). Structures were solved by SHELXT [23] and refined by full-matrix least-squares treatment against |F|^2^ in anisotropic approximation with SHELX 2014/7 [24] in ShelXle program [25]. *H*-atoms were refined in the geometrically calculated positions. The crystallographic data have been deposed in the Cambridge Crystallographic Data Centre under the deposition codes CCDC 2174852–2174855. These data can be obtained free of charge via https://www.ccdc.cam.ac.uk/data_request/cif, or by emailing data_request@ccdc.cam.ac.uk.

### 2.6. Raman Spectroscopy

Raman spectra were collected using a LabRAM HR Evolution (Horiba) spectrometer with the excitation by the 633 nm line of the He-Ne laser. The spectra at room temperatures were obtained in the backscattering geometry with a Raman microscope. The laser beam was focused to a diameter of 2 μm using a LMPlan FL 50×/0.50 Olympus objective. The spectral resolution was 0.7 cm^−1^. The laser power on the sample surface was about 0.03 mW.

### 2.7. Powder X-ray Diffractometry (PXRD)

XRD analysis of polycrystals was performed on Shimadzu XRD-7000 diffractometer (CuK-alpha radiation, Ni-filter, linear One Sight detector, 0.0143° 2θ step, 2 s per step). Plotting of PXRD patterns and data treatment was performed using X’Pert Plus software (see Supplementary Materials).

### 2.8. Thermogravimetric Analysis (TGA) and Computational Details

Details are given in Supplementary Materials.

## 3. Results and Discussion

Despite the general scheme of the preparation of polyhalogen-halometalates being rather simple (source of metal + source of halide, commonly taken as halide salt of organic cation + dihalogen), there are variations based on the choice of solvent. In some cases [7], ionic liquids were successfully used (this is a common approach in the synthesis of non-conventional polyhalogens [26,27,28,29,30]) for this purpose; additionally, organic solvents can be utilized. However, since we earlier found that combination of aqueous hydrohalic acids and metal oxide gives good results for M = Bi, Te, Sb, Sn, etc. [14,19], we decided to follow the same scheme for Se (SeO_2_ + concentrated aqueous HBr + Br_2_ + bromide salt of organic cation, see Section 2 for details). For **1**–**4**, it yields the formation of single crystals suitable for X-ray diffractometry.

**Figure 1:**
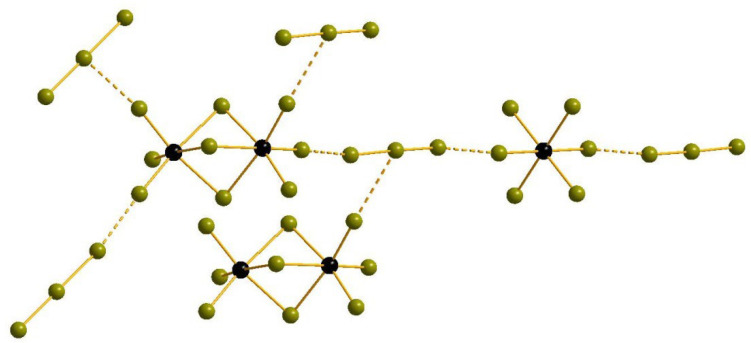
Br···Br non-covalent interactions in the structure of **1**. Here and below: Se, black; Br, olive-green; non-covalent contacts, dashed.

The structural data for **1**–**4** demonstrate that Se(IV) can form the complexes similar to those with Te(IV), but this occurs not in all cases—Se(IV) derivatives are prominently more diverse in terms of supramolecular chemistry. In the structure of **1**, there are two types of bromoselenate(IV) anions in the structure–mononuclear [SeBr_6_]^2−^ (Se-Br = 2.569–2.580 Å) and binuclear [Se_2_Br_9_]^-^. The latter type (two octahedra joint via one shared face) is very common for halometalates of Sb and Bi [31,32,33,34,35,36,37,38] but rare for Se [39,40]. The Sb-Br_term_ bonds are 2.374–2.406 Å, while the Sb-μ_2_-Br distances are expectedly longer (2.824–2.913 Å). The longest Se···Br interactions (2.913 Å) can also be regarded as strong chalcogen bonding, as follows from DFT calculations (see below). The tribromide anion is asymmetric (Br-Br = 2.467 and 2.668 Å). The system of Br···Br non-covalent interactions (assuming their presence for the distances lesser than the sum of Bondi’s van der Waals radii [41,42] for two Br atoms) is non-trivial. Each tribromide unit forms contacts with one [SeBr_6_]^2−^ (via terminal Br; Br···Br = 3.432 Å) and two [Se_2_Br_9_]^-^ anions (via central and terminal Br; Br···Br = 3.473 and 3.156 Å, respectively). Each [Se_2_Br_9_]^-^ interacts therefore with four Br_3_^-^ (Figure 1). Interestingly, the same composition of the anionic part was found in the salt described by Boyle et al. nearly two decades ago [40], but the system of non-covalent interactions in that case is not similar to the one in **1**.

**Figure 2:**
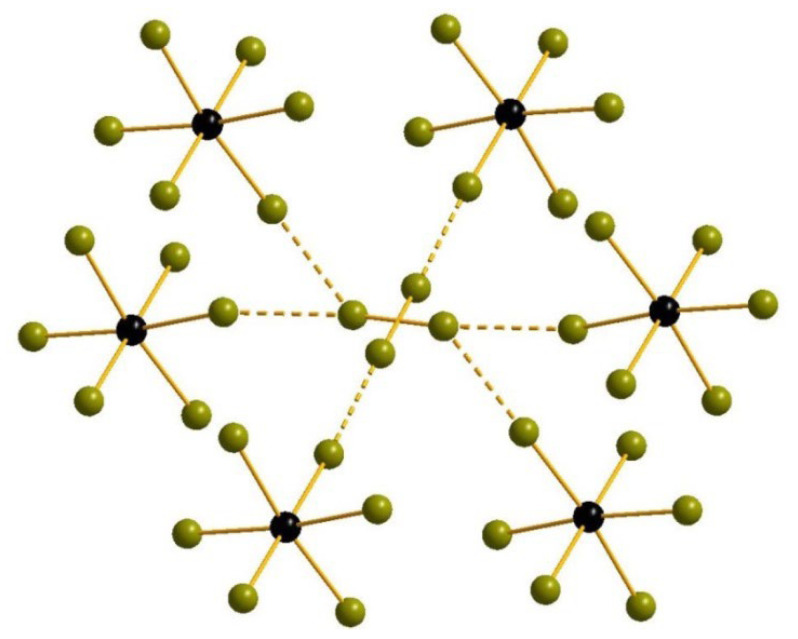
Br···Br interactions in the structure of 2. Only one position of disordered bromide ligands is shown.

In [SeBr_6_]^2−^ anions (Se-Br = 2.307–2.686 Å) in **2**, two bromide ligands are disordered over two positions each with 0.5 occupancy. The Br_2_ units are disordered as well (0.5:0.5, Br-Br = 2.328–2.351 Å). The composition of **2** is very similar to most common for polybromo-bromotellurates(IV) [15] (one Br_2_ per one octahedral [MBr_6_]^2-^), but the system of Br···Br non-covalent interactions is more sophisticated. For one of Br_2_ units, there is only one type of contacts which has the pattern identical to one found in polybromo-bromotellurates(IV)—it links bromoselenate(IV) anions and Br_2_ (Br···Br = 3.110 Å) into 1D linear chains with Br_term_-Br_Br2_-Br_Br2_ angle close to 180° (170.32°). For another Br_2_ position, there are two types of interactions—one is the same as described above (Br···Br = 3.289 Å, Br-Br-Br = 174.52°), while another involves the bromide ligands of [SeBr_6_]^2−^ of neighboring layer in packing (Br···Br = 3.359 Å) so that the Br-Br-Br angles are lower (111.74°) and a pseudo-3D structure is formed (Figure 2).

**Figure 3:**
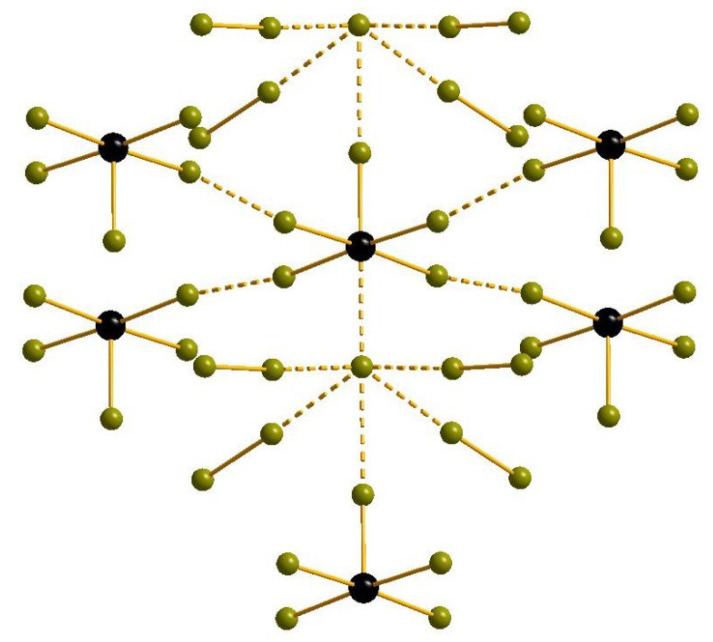
The system of non-covalent interactions in the structure of **3.**

The structure of **3** (Figure 3) features the presence of pyramidal [SeBr_5_]^−^ anions (Se-Br_eq_ and Se-Br_ax_ = 2.559 and 2.360 Å, respectively). Additionally, there are bromide anions which make coordination environment of Se pseudo-octahedral, but the Se···Br distances are too long for conventional covalent bonds (3.049 Å). Each of these Br^−^ interacts with four Br_2_ (Br-Br = 2.359 Å) units (Br···Br = 3.131 Å) and, additionally, with an axial bromide ligand of neighboring [SeBr_5_]^−^ (Br···Br = 3.238 Å). An interesting feature of this structure is that the Br···Br distances between the equatorial Br ligands of [SeBr_5_]^−^ anions are also shorter than the sum of van der Waals radii (3.225 Å). Very similar effect was previously described for bromoantimonates(V) [16], where the system of such hypothetic interactions can be 1D, 2D, or even 3D. It is assumed that those can be responsible for enhanced photophysical behavior of some Cat[SbBr_6_] salts, which were utilized as light absorbers in model solar cells [43].

**Figure 4:**
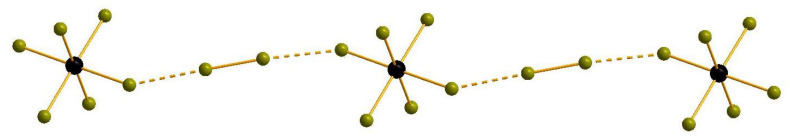
The system of non-covalent interactions in the structure of **4.**

Finally, complex **4** is isostructural to the tellurium-containing one-(1-MePy)_2_{[TeBr_6_](Br_2_)} [15]. The [SeBr_6_]^2−^ (Se-Br = 2.513–2.610 Å) and Br_2_ (Br-Br = 2.335 Å) units are linked (Br···Br = 3.143 Å, Br-Br-Br = 175.55°) into 1D linear chains (Figure 4).

**Figure 5:**
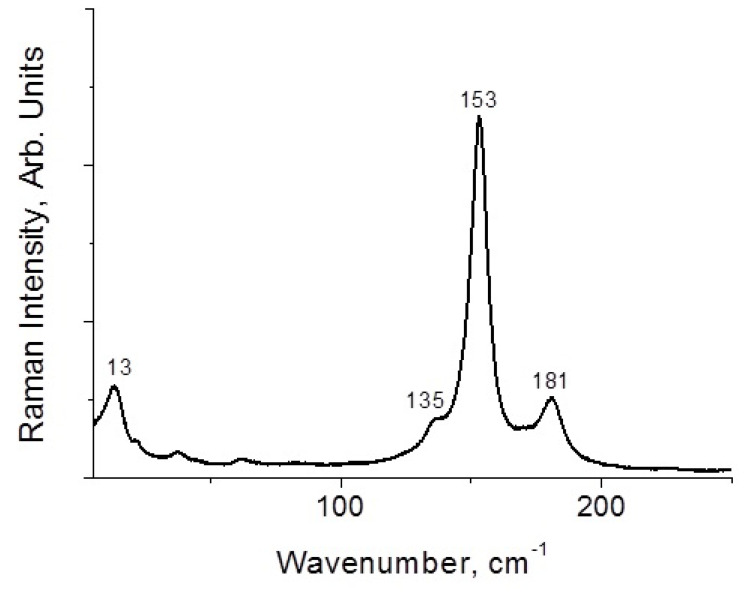
Raman spectrum of **1.**

**Figure 6:**
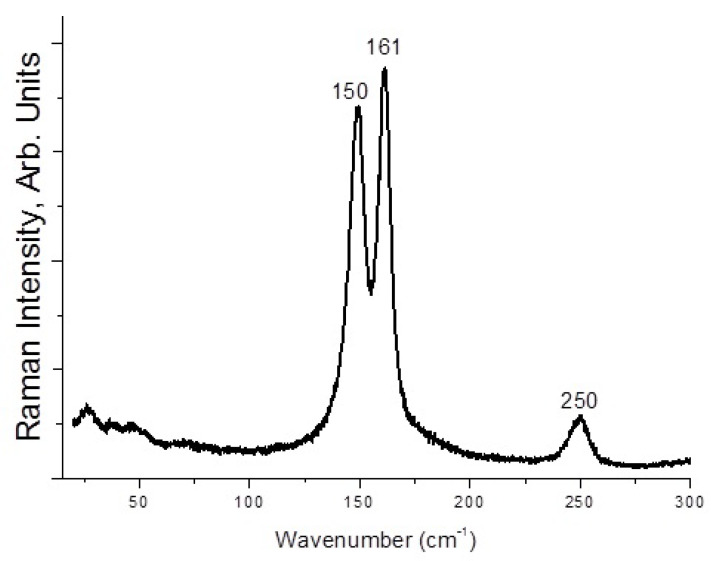
Raman spectrum of **2.**

**Figure 7:**
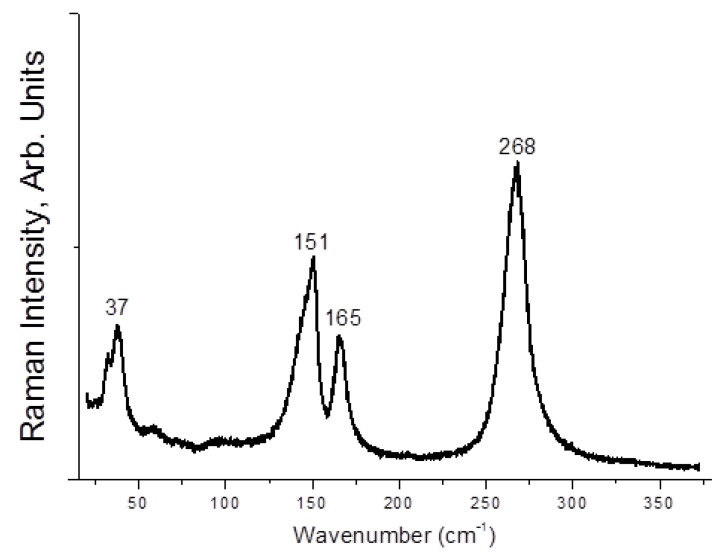
Raman spectrum of **4.**

**Figure 8:**
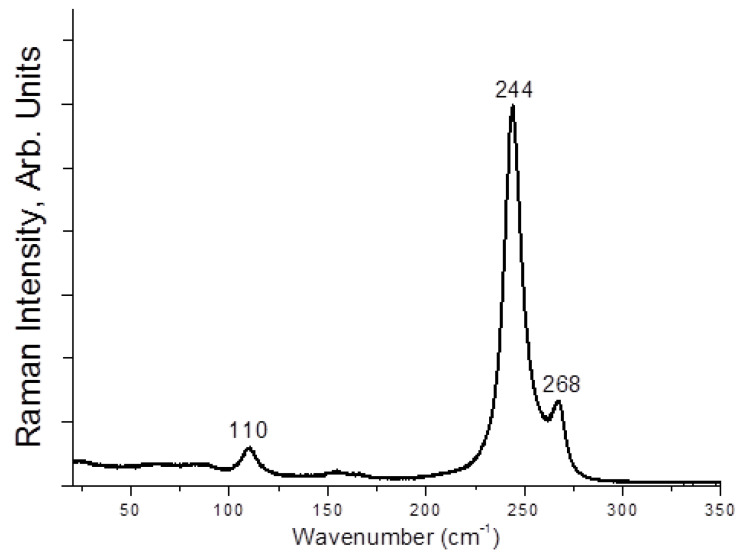
Raman spectrum of **3.**

Here, **1**–**4** demonstrate different stability while being kept outside the dibromine-containing mother liquor. Only **1** and **4** are stable enough for PXRD experiment (Figures S1 and S2); **1** is slightly contaminated by an unidentified minor byproduct, while **4** precipitates as a single phase. Moreover, **2** and **3** undergo decomposition with loss of Br_2_ (detectable visually). However, we applied Raman spectroscopy for freshly isolated solids. In the case of **1** (Figure 5), the bands corresponding to asymmetric tribromide anion must appear in the 150–160 and 180–190 ranges [44,45], so those are likely overlapped by bromoselenate (150–180 cm^−1^) [46,47,48]. The bands of latter appear in spectrum of **2** at 150 and 161 cm^−1^ (Figure 6), while Br_2_ has highly characteristic mode at 250 cm^−1^ (for Te(IV) derivatives [49], this band is at 265–271 cm^−1^). For **4**, the spectrum is very similar (Figure 7), but the Br_2_ band appears at higher wavelength (268 cm^−1^). Finally, **3** has the least trivial spectrum (Figure 8). We assume that the band at 110 cm^−1^ corresponds to [SeBr_5_]^−^ anion, while strong bands at 244 and 268 cm^−1^ are related to different Br_2_ units in the structure.

For theoretical investigation of the nature of non-covalent interactions in the abovementioned complexes, we followed the approach which was extensively used by us for the examination of other relevant supramolecular systems: DFT calculations for non-optimized structures [50,51,52,53,54] (atomic coordinates extracted from XRD data) and QTAIM analysis [55] of electron density distribution. Unfortunately, disordering in the structure of **2** did not allow to perform analysis for this compound. For **1**, **3,** and **4**, results are summarized in Table 1 (see Supplementary Materials for graphical representation). Several interesting observations can be made. First, the energies of Br···Br interactions between the [SbBr_5_]^-^ units in **3** are comparable with those of contacts with polyhalogen units, and this is very similar to the situation in polybromo-bromoantimonates(V) [16]. Most likely, this feature is general for halometalates with a high charge of the central atom. Second, the Se···Br interaction in **3** is truly non-covalent (-G(r)/V(r) ≥ 1) [56], so it must be considered as chalcogen bonding. Third, all interactions in abovementioned structures are attractive [57,58]. The energies (up to 3.6 kcal/mol for Br···Br) are within the ranges typical for the compounds of this family.

Since PXRD (see Supplementary Materials) indicates the permanent presence of minor impurities in **1** and since **2** and **3** are, as mentioned above, unstable, we performed TGA only for the last complex. Results are summarized in Supplementary Materials (Figure S3); thermal decomposition corresponding to the loss of dibromine unit (this pathway is common for the complexes of this sort) occurs at > 100 °C, followed by complete destruction with total mass loss at >250 °C.

## 4. Conclusions

We demonstrated that selenium can form extensive family of polybromo-bromoselenates (IV), which can be structurally different from corresponding Te (IV) derivatives. The nature of this element enables formation of non-covalent interactions between the bromide ligands of neighboring [SeBr_5_]^−^ units, similar to how it occurs in Sb (V) complexes. Besides, unlike Te (IV), selenium can participate in formation of chalcogen bonding in these compounds.

## Figures and Tables

**Table 1:** Values of the density of all electrons ρ(**r**), Laplacian of electron density ∇^2^ρ(**r**) and appropriate λ_2_ eigenvalues, energy density H_b_, potential energy density V(**r**), and Lagrangian kinetic energy G(**r**), electron localization function ELF (a.u.) at the bond critical points (3, –1) for intermolecular interactions in **1**, **3**, **4**, and their estimated strength E_int_ (kcal/mol).

Contact	% of Σ (vdW radii)	ρ(r)	∇^2^ρ(r)	−λ_2_	H_b_	−V(r)	G(r)	ELF	E_int_^a^
1									
Br···Br 3.432 Å	94	0.011	0.027	0.011	0.000	0.006	0.006	0.053	2.2
Br···Br 3.156 Å	86	0.017	0.044	0.017	0.001	0.009	0.010	0.094	3.3
Br···Br 3.473 Å	95	0.011	0.028	0.011	0.001	0.006	0.007	0.047	2.2
Se···Br 2.913 Å	78	0.031	0.080	0.031	0.001	0.021	0.020	0.157	7.6
**3**									
Se···Br 3.049 Å	82	0.024	0.052	0.024	0.001	0.012	0.013	0.175	4.4
Br···Br 3.225 Å	88	0.015	0.041	0.015	0.001	0.009	0.010	0.074	3.3
Br···Br 3.238 Å	88	0.016	0.036	0.016	0.000	0.008	0.008	0.111	2.9
Br···Br 3.131 Å	86	0.020	0.046	0.020	0.001	0.010	0.011	0.131	3.6
**4**									
Br···Br 3.143 Å	86	0.019	0.042	0.019	0.001	0.009	0.010	0.133	3.3

^a^ E_int_ = 0.58(−V(r)) (this empirical correlation between the interaction energy and the potential energy density of electrons at the bond critical points (3, −1) was specifically developed for non-covalent interactions involving bromine atoms) [59].

## Data Availability

Not applicable.

## Supplementary Materials

The following supporting information can be downloaded at: https://www.mdpi.com/article/10.3390/molecules27165355/s1, XRD, PXRD, and TGA data, as well as computational details.

## Sample Availability

Samples of the compounds are not available from the authors.
