# Supplementary material for: Selenium(IV) Polybromide Complexes: Structural Diversity Driven by Halogen and Chalcogen Bonding
Source: Molecules. 2022 Aug 22;27(16):5355. doi: 10.3390/molecules27165355 (PMC9415170; doi:10.3390/molecules27165355)

checkCIF/PLATON report

Structure factors have been supplied for datablock(s) 1

THIS REPORT IS FOR GUIDANCE ONLY. IF USED AS PART OF A REVIEW PROCEDURE FOR PUBLICATION, IT SHOULD NOT REPLACE THE EXPERTISE OF AN EXPERIENCED CRYSTALLOGRAPHIC REFEREE.

No syntax errors found.   CIF dictionary   Interpreting this report

Datablock: 1

Bond precision:	C-C = 0.0163 A		Wavelength=0.71073
Cell:	a=11.0372(9)	b=16.0823(12)	c=33.061(3)
	alpha=90	beta=94.534(3)	gamma=90
Temperature:	150 K		

	Calculated	Reported
Volume	5850.1(8)	5850.1(8)
Space group	C 2/c	C 2/c
Hall group	-C 2yc	-C 2yc
Moiety formula	Br9 Se2, Br6 Se, 5(C6 H8 N), 2(Br3)	?
Sum formula	C30 H40 Br21 N5 Se3	C30 H40 Br21 N5 Se3
Mr	2385.45	2385.66
Dx,g cm-3	2.708	2.709
Z	4	4
Mu (mm-1)	16.259	16.259
F000	4368.0	4368.0
F000'	4348.40	
h,k,lmax	14,21,43	14,21,43
Nref	6995	6980
Tmin,Tmax	0.265,0.522	0.452,0.746
Tmin'	0.223	

Correction method= # Reported T Limits: Tmin=0.452 Tmax=0.746
AbsCorr = MULTI-SCAN

Data completeness= 0.998   Theta(max)= 27.905

R(reflections)= 0.0625(5748)   wR2(reflections)= 0.1524(6980)

S = 1.305   Npar= 253

The following ALERTS were generated. Each ALERT has the format
test-name_ALERT_alert-type_alert-level.
Click on the hyperlinks for more details of the test.

Alert level B

```
PLAT341_ALERT_3_B Low Bond Precision on  C-C Bonds ...............     0.0163 Ang.
PLAT434_ALERT_2_B Short Inter HL..HL Contact Br6       ..Br7      .       3.16 Ang.
                                                     x,y,z  =      1_555 Check
```

Alert level C

```
PLAT790_ALERT_4_C Centre of Gravity not Within Unit Cell: Resd.  #          1 Note
              Br9 Se2
PLAT906_ALERT_3_C Large K Value in the Analysis of Variance ......     12.264 Check
PLAT906_ALERT_3_C Large K Value in the Analysis of Variance ......      2.701 Check
PLAT911_ALERT_3_C Missing FCF Refl Between Thmin & STh/L=    0.600          2 Report
PLAT972_ALERT_2_C Check Calcd Resid. Dens.  0.96Ang From Br11          -1.74 eA-3
PLAT977_ALERT_2_C Check Negative Difference Density on H3         .     -0.42 eA-3
PLAT977_ALERT_2_C Check Negative Difference Density on H8         .     -0.47 eA-3
PLAT977_ALERT_2_C Check Negative Difference Density on H9A        .     -0.63 eA-3
```

Alert level G

```
PLAT003_ALERT_2_G Number of Uiso or Uij Restrained non-H Atoms ...          3 Report
PLAT007_ALERT_5_G Number of Unrefined Donor-H Atoms .............          3 Report
PLAT083_ALERT_2_G SHELXL Second Parameter in WGHT  Unusually Large     216.77 Why ?
PLAT178_ALERT_4_G The CIF-Embedded .res File Contains SIMU Records          1 Report
PLAT300_ALERT_4_G Atom Site Occupancy of N3         Constrained at        0.5 Check
PLAT300_ALERT_4_G Atom Site Occupancy of C13        Constrained at        0.5 Check
PLAT300_ALERT_4_G Atom Site Occupancy of C14        Constrained at        0.5 Check
PLAT300_ALERT_4_G Atom Site Occupancy of C15        Constrained at        0.5 Check
PLAT300_ALERT_4_G Atom Site Occupancy of C16        Constrained at        0.5 Check
PLAT300_ALERT_4_G Atom Site Occupancy of C17        Constrained at        0.5 Check
PLAT300_ALERT_4_G Atom Site Occupancy of C18        Constrained at        0.5 Check
PLAT300_ALERT_4_G Atom Site Occupancy of H3         Constrained at        0.5 Check
PLAT300_ALERT_4_G Atom Site Occupancy of H13        Constrained at        0.5 Check
PLAT300_ALERT_4_G Atom Site Occupancy of H14        Constrained at        0.5 Check
PLAT300_ALERT_4_G Atom Site Occupancy of H16        Constrained at        0.5 Check
PLAT300_ALERT_4_G Atom Site Occupancy of H17        Constrained at        0.5 Check
PLAT300_ALERT_4_G Atom Site Occupancy of H18A       Constrained at        0.5 Check
PLAT300_ALERT_4_G Atom Site Occupancy of H18B       Constrained at        0.5 Check
PLAT300_ALERT_4_G Atom Site Occupancy of H18C       Constrained at        0.5 Check
PLAT302_ALERT_4_G Anion/Solvent/Minor-Residue Disorder (Resd  5  )       100% Note
PLAT304_ALERT_4_G Non-Integer Number of Atoms in ..... (Resd  5  )       7.50 Check
PLAT434_ALERT_2_G Short Inter HL..HL Contact Br2       ..Br4      .       3.43 Ang.
                                                     x,y,z  =      1_555 Check
PLAT434_ALERT_2_G Short Inter HL..HL Contact Br5       ..Br8      .       3.47 Ang.
                                             1/2+x,1/2+y,z  =      3_555 Check
PLAT789_ALERT_4_G Atoms with Negative _atom_site_disorder_group  #         15 Check
PLAT860_ALERT_3_G Number of Least-Squares Restraints .............         12 Note
PLAT883_ALERT_1_G No Info/Value for _atom_sites_solution_primary .     Please Do !
PLAT910_ALERT_3_G Missing # of FCF Reflection(s) Below Theta(Min).          1 Note
PLAT912_ALERT_4_G Missing # of FCF Reflections Above STh/L=  0.600         14 Note
PLAT913_ALERT_3_G Missing # of Very Strong Reflections in FCF ....          1 Note
```

```
PLAT933_ALERT_2_G Number of HKL-OMIT Records in Embedded .res File          1 Note
PLAT961_ALERT_5_G Dataset Contains no Negative Intensities .......     Please Check
PLAT965_ALERT_2_G The SHELXL WEIGHT Optimisation has not Converged     Please Check
PLAT978_ALERT_2_G Number C-C Bonds with Positive Residual Density.          0 Info
```

```
   0 ALERT level A = Most likely a serious problem - resolve or explain
   2 ALERT level B = A potentially serious problem, consider carefully
   8 ALERT level C = Check. Ensure it is not caused by an omission or oversight
  33 ALERT level G = General information/check it is not something unexpected

   1 ALERT type 1 CIF construction/syntax error, inconsistent or missing data
  12 ALERT type 2 Indicator that the structure model may be wrong or deficient
   7 ALERT type 3 Indicator that the structure quality may be low
  21 ALERT type 4 Improvement, methodology, query or suggestion
   2 ALERT type 5 Informative message, check
```

It is advisable to attempt to resolve as many as possible of the alerts in all categories. Often the minor alerts point to easily fixed oversights, errors and omissions in your CIF or refinement strategy, so attention to these fine details can be worthwhile. In order to resolve some of the more serious problems it may be necessary to carry out additional measurements or structure refinements. However, the purpose of your study may justify the reported deviations and the more serious of these should normally be commented upon in the discussion or experimental section of a paper or in the "special_details" fields of the CIF. checkCIF was carefully designed to identify outliers and unusual parameters, but every test has its limitations and alerts that are not important in a particular case may appear. Conversely, the absence of alerts does not guarantee there are no aspects of the results needing attention. It is up to the individual to critically assess their own results and, if necessary, seek expert advice.

Publication of your CIF in IUCr journals

A basic structural check has been run on your CIF. These basic checks will be run on all CIFs submitted for publication in IUCr journals (*Acta Crystallographica*, *Journal of Applied Crystallography*, *Journal of Synchrotron Radiation*); however, if you intend to submit to *Acta Crystallographica Section C* or *E* or *IUCrData*, you should make sure that full publication checks are run on the final version of your CIF prior to submission.

Publication of your CIF in other journals

Please refer to the *Notes for Authors* of the relevant journal for any special instructions relating to CIF submission.

PLATON version of 18/05/2022; check.def file version of 17/05/2022

Datablock 1 - ellipsoid plot

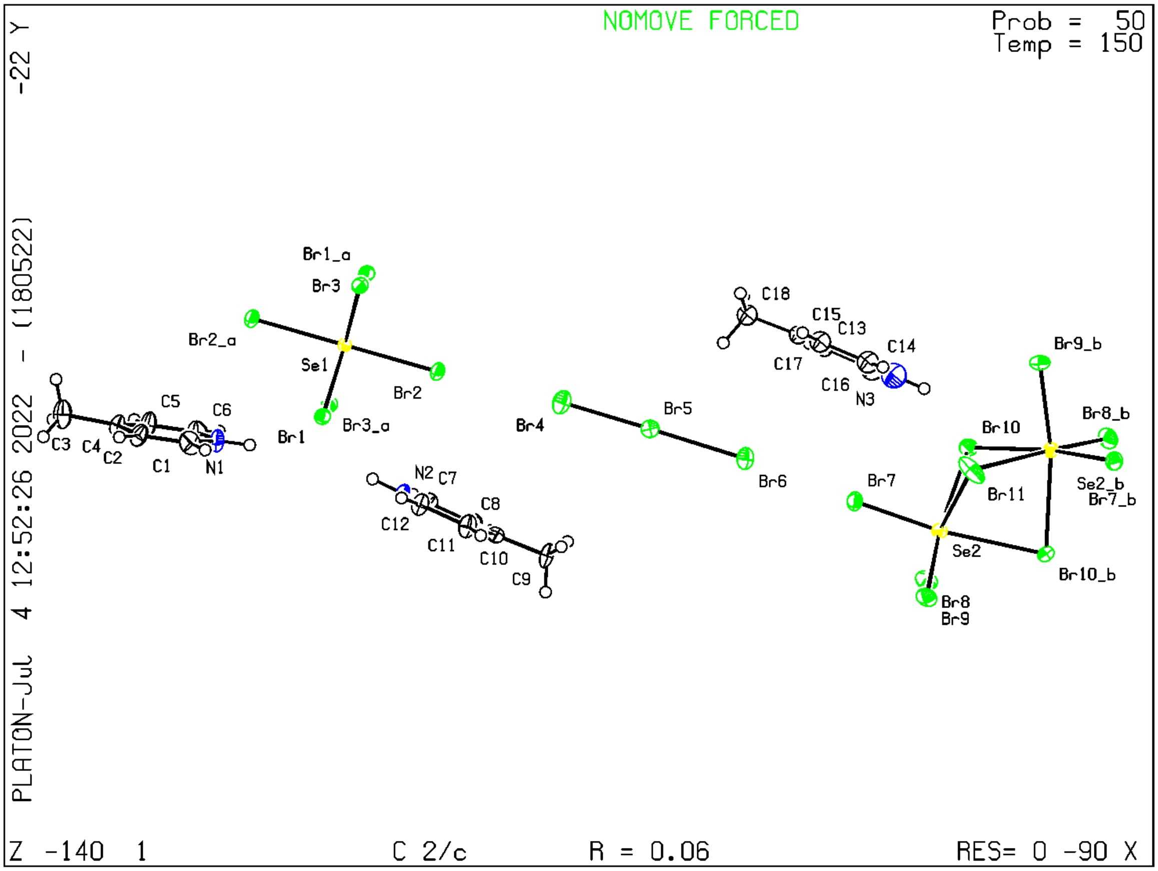

Supplement: Supplementary file 1 [file molecules-27-05355-s001.zip › checkcif 1.pdf]
