# Supplementary material for: Selenium(IV) Polybromide Complexes: Structural Diversity Driven by Halogen and Chalcogen Bonding
Source: Molecules. 2022 Aug 22;27(16):5355. doi: 10.3390/molecules27165355 (PMC9415170; doi:10.3390/molecules27165355)

checkCIF/PLATON report

Structure factors have been supplied for datablock(s) 2

THIS REPORT IS FOR GUIDANCE ONLY. IF USED AS PART OF A REVIEW PROCEDURE FOR PUBLICATION, IT SHOULD NOT REPLACE THE EXPERTISE OF AN EXPERIENCED CRYSTALLOGRAPHIC REFEREE.

No syntax errors found. CIF dictionary Interpreting this report

Datablock: 2

Bond precision: C-C = 0.0174 A Wavelength=0.71073

Cell:	a=17.7961(15)	b=7.4754(6)	c=17.6525(17)
	alpha=90	beta=100.133(5)	gamma=90
Temperature:	150 K		

	Calculated	Reported
Volume	2311.7(4)	2311.7(4)
Space group	C 2/c	C 2/c
Hall group	-C 2yc	-C 2yc
Moiety formula	Br6 Se, 2(C6 H8 N), 2(Br)	?
Sum formula	C12 H16 Br8 N2 Se	C12 H16 Br8 N2 Se
Mr	906.43	906.51
Dx,g cm-3	2.604	2.605
Z	4	4
Mu (mm-1)	15.451	15.451
F000	1664.0	1664.0
F000'	1656.53	
h,k,lmax	22,9,22	22,9,22
Nref	2564	2561
Tmin,Tmax	0.236,0.291	0.540,0.746
Tmin'	0.205	

Correction method= # Reported T Limits: Tmin=0.540 Tmax=0.746
AbsCorr = MULTI-SCAN

Data completeness= 0.999 Theta(max)= 27.140

R(reflections)= 0.0464(1919) wR2(reflections)= 0.1250(2561)

S = 1.093 Npar= 124

The following ALERTS were generated. Each ALERT has the format
test-name_ALERT_alert-type_alert-level.
Click on the hyperlinks for more details of the test.

Alert level B

```
PLAT230_ALERT_2_B Hirshfeld Test Diff for    Br1      --Se1      .       10.1 s.u.
PLAT230_ALERT_2_B Hirshfeld Test Diff for    Br2      --Se1      .       17.1 s.u.
PLAT341_ALERT_3_B Low Bond Precision on  C-C Bonds ..............     0.0174 Ang.
```

Alert level C

```
PLAT242_ALERT_2_C Low    'MainMol' Ueq as Compared to Neighbors of         N1 Check
PLAT250_ALERT_2_C Large U3/U1 Ratio for Average U(i,j) Tensor ....        2.8 Note
PLAT362_ALERT_2_C Short  C(sp3)-C(sp2) Bond  C7       - C8       .       1.38 Ang.
PLAT906_ALERT_3_C Large K Value in the Analysis of Variance ......      5.753 Check
PLAT911_ALERT_3_C Missing FCF Refl Between Thmin & STh/L=    0.600          3 Report
```

Alert level G

```
PLAT003_ALERT_2_G Number of Uiso or Uij Restrained non-H Atoms ...          6 Report
PLAT007_ALERT_5_G Number of Unrefined Donor-H Atoms .............           1 Report
PLAT083_ALERT_2_G SHELXL Second Parameter in WGHT  Unusually Large      18.29 Why ?
PLAT178_ALERT_4_G The CIF-Embedded .res File Contains SIMU Records          1 Report
PLAT230_ALERT_2_G Hirshfeld Test Diff for    Br5A     --Se1      .       20.9 s.u.
PLAT230_ALERT_2_G Hirshfeld Test Diff for    Br5B     --Se1      .       22.2 s.u.
PLAT300_ALERT_4_G Atom Site Occupancy of Br5A       Constrained at        0.5 Check
PLAT300_ALERT_4_G Atom Site Occupancy of Br5B       Constrained at        0.5 Check
PLAT300_ALERT_4_G Atom Site Occupancy of Br7        Constrained at        0.5 Check
PLAT300_ALERT_4_G Atom Site Occupancy of Br8        Constrained at        0.5 Check
PLAT301_ALERT_3_G Main Residue  Disorder .............(Resd  1  )        29% Note
PLAT302_ALERT_4_G Anion/Solvent/Minor-Residue Disorder (Resd  3  )       100% Note
PLAT302_ALERT_4_G Anion/Solvent/Minor-Residue Disorder (Resd  4  )       100% Note
PLAT304_ALERT_4_G Non-Integer Number of Atoms in ..... (Resd  3  )       0.50 Check
PLAT304_ALERT_4_G Non-Integer Number of Atoms in ..... (Resd  4  )       0.50 Check
PLAT434_ALERT_2_G Short Inter HL..HL Contact Br1      ..Br8      .       3.10 Ang.
                                                      x,y,z  =      1_555 Check
PLAT434_ALERT_2_G Short Inter HL..HL Contact Br2      ..Br7      .       3.29 Ang.
                                          1/2-x,-1/2+y,1/2-z  =      4_545 Check
PLAT764_ALERT_4_G Overcomplete CIF Bond List Detected (Rep/Expd) .       1.33 Ratio
PLAT779_ALERT_4_G Suspect or Irrelevant (Bond) Angle(s) in CIF ...      11.82 Deg.
              BR5B -SE1  -BR5A    2_555   1_555   2_555 ......     #    9 Check
PLAT779_ALERT_4_G Suspect or Irrelevant (Bond) Angle(s) in CIF ...      11.82 Deg.
              BR5B -SE1  -BR5A    1_555   1_555   1_555 ......     #   14 Check
PLAT779_ALERT_4_G Suspect or Irrelevant (Bond) Angle(s) in CIF ...      42.74 Deg.
              BR8  -BR7  -BR7     1_555   1_555   7_565 ......     #   57 Check
PLAT779_ALERT_4_G Suspect or Irrelevant (Bond) Angle(s) in CIF ...      43.25 Deg.
              BR7  -BR8  -BR8     1_555   1_555   7_565 ......     #   60 Check
PLAT860_ALERT_3_G Number of Least-Squares Restraints ............          36 Note
PLAT883_ALERT_1_G No Info/Value for _atom_sites_solution_primary .     Please Do !
PLAT912_ALERT_4_G Missing # of FCF Reflections Above STh/L=  0.600          1 Note
PLAT913_ALERT_3_G Missing # of Very Strong Reflections in FCF ....          2 Note
PLAT933_ALERT_2_G Number of HKL-OMIT Records in Embedded .res File          2 Note
PLAT961_ALERT_5_G Dataset Contains no Negative Intensities .......     Please Check
PLAT965_ALERT_2_G The SHELXL WEIGHT Optimisation has not Converged     Please Check
```

```
PLAT978_ALERT_2_G Number C-C Bonds with Positive Residual Density.          0 Info
```

```
   0 ALERT level A = Most likely a serious problem - resolve or explain
   3 ALERT level B = A potentially serious problem, consider carefully
   5 ALERT level C = Check. Ensure it is not caused by an omission or oversight
  30 ALERT level G = General information/check it is not something unexpected

   1 ALERT type 1 CIF construction/syntax error, inconsistent or missing data
  14 ALERT type 2 Indicator that the structure model may be wrong or deficient
   6 ALERT type 3 Indicator that the structure quality may be low
  15 ALERT type 4 Improvement, methodology, query or suggestion
   2 ALERT type 5 Informative message, check
```

It is advisable to attempt to resolve as many as possible of the alerts in all categories. Often the minor alerts point to easily fixed oversights, errors and omissions in your CIF or refinement strategy, so attention to these fine details can be worthwhile. In order to resolve some of the more serious problems it may be necessary to carry out additional measurements or structure refinements. However, the purpose of your study may justify the reported deviations and the more serious of these should normally be commented upon in the discussion or experimental section of a paper or in the "special_details" fields of the CIF. checkCIF was carefully designed to identify outliers and unusual parameters, but every test has its limitations and alerts that are not important in a particular case may appear. Conversely, the absence of alerts does not guarantee there are no aspects of the results needing attention. It is up to the individual to critically assess their own results and, if necessary, seek expert advice.

Publication of your CIF in IUCr journals

A basic structural check has been run on your CIF. These basic checks will be run on all CIFs submitted for publication in IUCr journals (*Acta Crystallographica*, *Journal of Applied Crystallography*, *Journal of Synchrotron Radiation*); however, if you intend to submit to *Acta Crystallographica Section C* or *E* or *IUCrData*, you should make sure that full publication checks are run on the final version of your CIF prior to submission.

Publication of your CIF in other journals

Please refer to the *Notes for Authors* of the relevant journal for any special instructions relating to CIF submission.

PLATON version of 18/05/2022; check.def file version of 17/05/2022

Datablock 2 - ellipsoid plot

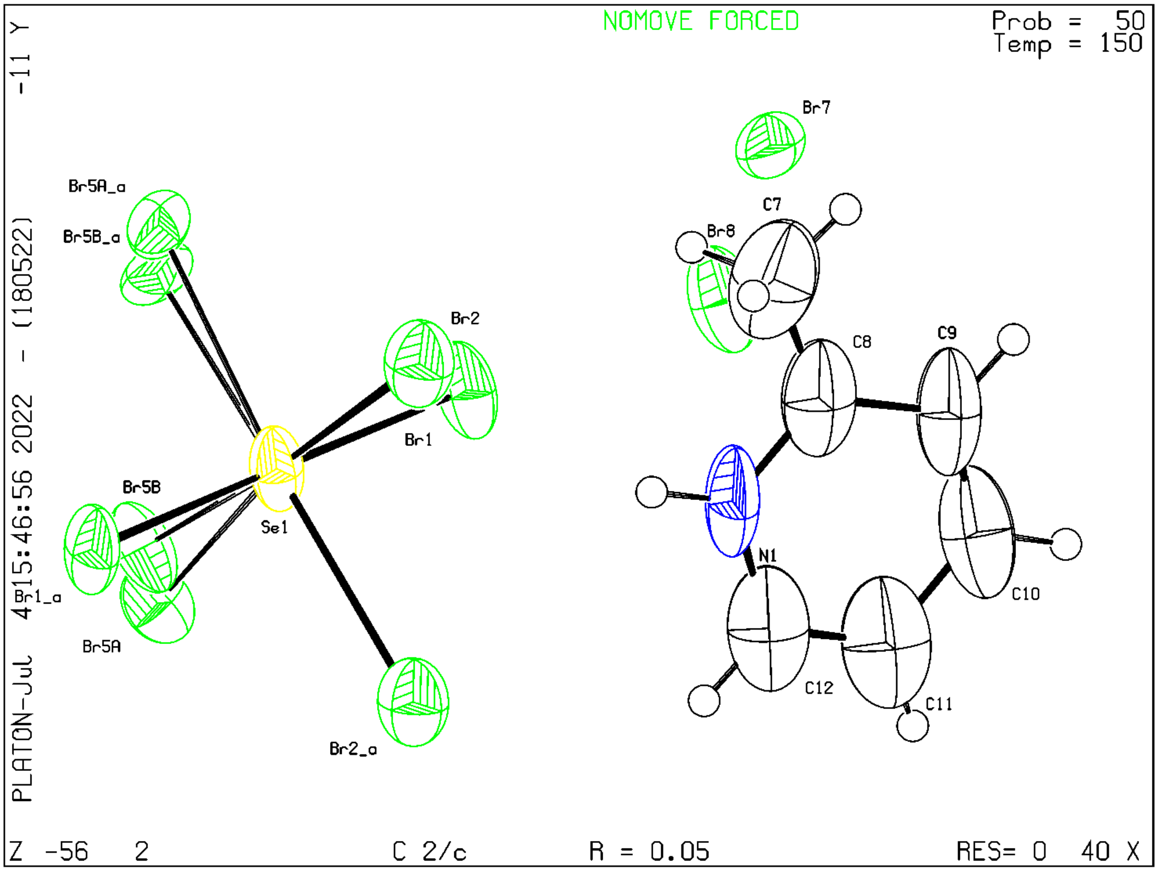

Supplement: Supplementary file 1 [file molecules-27-05355-s001.zip › checkcif 2.pdf]
