# Supplementary material for: Selenium(IV) Polybromide Complexes: Structural Diversity Driven by Halogen and Chalcogen Bonding
Source: Molecules. 2022 Aug 22;27(16):5355. doi: 10.3390/molecules27165355 (PMC9415170; doi:10.3390/molecules27165355)

checkCIF/PLATON report

Structure factors have been supplied for datablock(s) 3

THIS REPORT IS FOR GUIDANCE ONLY. IF USED AS PART OF A REVIEW PROCEDURE FOR PUBLICATION, IT SHOULD NOT REPLACE THE EXPERTISE OF AN EXPERIENCED CRYSTALLOGRAPHIC REFEREE.

No syntax errors found. CIF dictionary Interpreting this report

Datablock: 3

Bond precision:	C-C = 0.0050 A	Wavelength=0.71073	
Cell:	a=11.7570(5)	b=11.7570(5)	c=8.6464(5)
	alpha=90	beta=90	gamma=90
Temperature:	188 K		

	Calculated	Reported
Volume	1195.17(12)	1195.17(12)
Space group	P 4/n m m	P 4/n m m
Hall group	-P 4a 2a	-P 4a 2a
Moiety formula	2(C5 H6 N), Br5 Se, 2(Br2), Br	?
Sum formula	C10 H12 Br10 N2 Se	C10 H12 Br10 N2 Se
Mr	1038.18	1038.28
Dx,g cm-3	2.885	2.885
Z	2	2
Mu (mm-1)	18.286	18.286
F000	940.0	940.0
F000’	935.45	
h,k,lmax	17,17,12	17,17,12
Nref	1157	1154
Tmin,Tmax	0.374,0.518	0.572,0.746
Tmin’	0.345	

Correction method= # Reported T Limits: Tmin=0.572 Tmax=0.746
AbsCorr = MULTI-SCAN

Data completeness= 0.997 Theta(max)= 31.497

R(reflections)= 0.0236(955) wR2(reflections)= 0.0481(1154)

S = 1.091 Npar= 42

The following ALERTS were generated. Each ALERT has the format
test-name_ALERT_alert-type_alert-level.
Click on the hyperlinks for more details of the test.

Alert level A

```
PLAT431_ALERT_2_A Short Inter HL..A Contact  Br3      ..Se1      .       3.05 Ang.
                                                    x,y,z   =      1_555 Check
```

Alert level B

```
PLAT434_ALERT_2_B Short Inter HL..HL Contact Br1      ..Br3      .       3.24 Ang.
                                                 x,y,-1+z   =      1_554 Check
PLAT434_ALERT_2_B Short Inter HL..HL Contact Br2      ..Br2      .       3.23 Ang.
                                              1-y,1-x,1-z   =      8_666 Check
PLAT434_ALERT_2_B Short Inter HL..HL Contact Br3      ..Br4      .       3.13 Ang.
                                                    x,y,z   =      1_555 Check
PLAT434_ALERT_2_B Short Inter HL..HL Contact Br3      ..Br4      .       3.13 Ang.
                                                3/2-y,x,z   =      3_655 Check
PLAT434_ALERT_2_B Short Inter HL..HL Contact Br3      ..Br4      .       3.13 Ang.
                                                y,3/2-x,z   =      4_565 Check
PLAT434_ALERT_2_B Short Inter HL..HL Contact Br3      ..Br4      .       3.13 Ang.
                                            3/2-x,3/2-y,z   =      2_665 Check
```

Alert level C

```
PLAT350_ALERT_3_C Short  C-H (X0.96,N1.08A)  C1       - H2       .       0.83 Ang.
PLAT420_ALERT_2_C D-H Bond Without Acceptor  N1       --H1A      .     Please Check
PLAT420_ALERT_2_C D-H Bond Without Acceptor  N1       --H1B      .     Please Check
PLAT906_ALERT_3_C Large K Value in the Analysis of Variance ......      3.791 Check
PLAT911_ALERT_3_C Missing FCF Refl Between Thmin & STh/L=    0.600          2 Report
```

Alert level G

```
PLAT164_ALERT_4_G Nr. of Refined C-H H-Atoms in Heavy-Atom Struct.          1 Note
PLAT168_ALERT_4_G The CIF-Embedded .res File Contains EXYZ Records          2 Report
PLAT171_ALERT_4_G The CIF-Embedded .res File Contains EADP Records          2 Report
PLAT300_ALERT_4_G Atom Site Occupancy of N1         Constrained at        0.5 Check
PLAT300_ALERT_4_G Atom Site Occupancy of C2         Constrained at        0.5 Check
PLAT300_ALERT_4_G Atom Site Occupancy of H1A        Constrained at        0.5 Check
PLAT300_ALERT_4_G Atom Site Occupancy of H1B        Constrained at        0.5 Check
PLAT301_ALERT_3_G Main Residue  Disorder ..............(Resd  1  )        50% Note
PLAT304_ALERT_4_G Non-Integer Number of Atoms in ..... (Resd  4  )       0.12 Check
PLAT779_ALERT_4_G Suspect or Irrelevant (Bond) Angle(s) in CIF ...       0.00 Deg.
              H1A  -C2   -H1B     1_555   1_555   1_555 ......     #     9 Check
PLAT883_ALERT_1_G No Info/Value for _atom_sites_solution_primary .     Please Do !
PLAT912_ALERT_4_G Missing # of FCF Reflections Above STh/L=  0.600          1 Note
PLAT913_ALERT_3_G Missing # of Very Strong Reflections in FCF ....          2 Note
PLAT933_ALERT_2_G Number of HKL-OMIT Records in Embedded .res File          2 Note
PLAT961_ALERT_5_G Dataset Contains no Negative Intensities .......     Please Check
PLAT965_ALERT_2_G The SHELXL WEIGHT Optimisation has not Converged     Please Check
PLAT978_ALERT_2_G Number C-C Bonds with Positive Residual Density.          0 Info
```

1 **ALERT level A** = Most likely a serious problem - resolve or explain

```
 6 ALERT level B = A potentially serious problem, consider carefully
 5 ALERT level C = Check. Ensure it is not caused by an omission or oversight
17 ALERT level G = General information/check it is not something unexpected

 1 ALERT type 1 CIF construction/syntax error, inconsistent or missing data
12 ALERT type 2 Indicator that the structure model may be wrong or deficient
 5 ALERT type 3 Indicator that the structure quality may be low
10 ALERT type 4 Improvement, methodology, query or suggestion
 1 ALERT type 5 Informative message, check
```

It is advisable to attempt to resolve as many as possible of the alerts in all categories. Often the minor alerts point to easily fixed oversights, errors and omissions in your CIF or refinement strategy, so attention to these fine details can be worthwhile. In order to resolve some of the more serious problems it may be necessary to carry out additional measurements or structure refinements. However, the purpose of your study may justify the reported deviations and the more serious of these should normally be commented upon in the discussion or experimental section of a paper or in the "special_details" fields of the CIF. checkCIF was carefully designed to identify outliers and unusual parameters, but every test has its limitations and alerts that are not important in a particular case may appear. Conversely, the absence of alerts does not guarantee there are no aspects of the results needing attention. It is up to the individual to critically assess their own results and, if necessary, seek expert advice.

Publication of your CIF in IUCr journals

A basic structural check has been run on your CIF. These basic checks will be run on all CIFs submitted for publication in IUCr journals (*Acta Crystallographica*, *Journal of Applied Crystallography*, *Journal of Synchrotron Radiation*); however, if you intend to submit to *Acta Crystallographica Section C* or *E* or *IUCrData*, you should make sure that full publication checks are run on the final version of your CIF prior to submission.

Publication of your CIF in other journals

Please refer to the *Notes for Authors* of the relevant journal for any special instructions relating to CIF submission.

PLATON version of 18/05/2022; check.def file version of 17/05/2022

Datablock 3 - ellipsoid plot

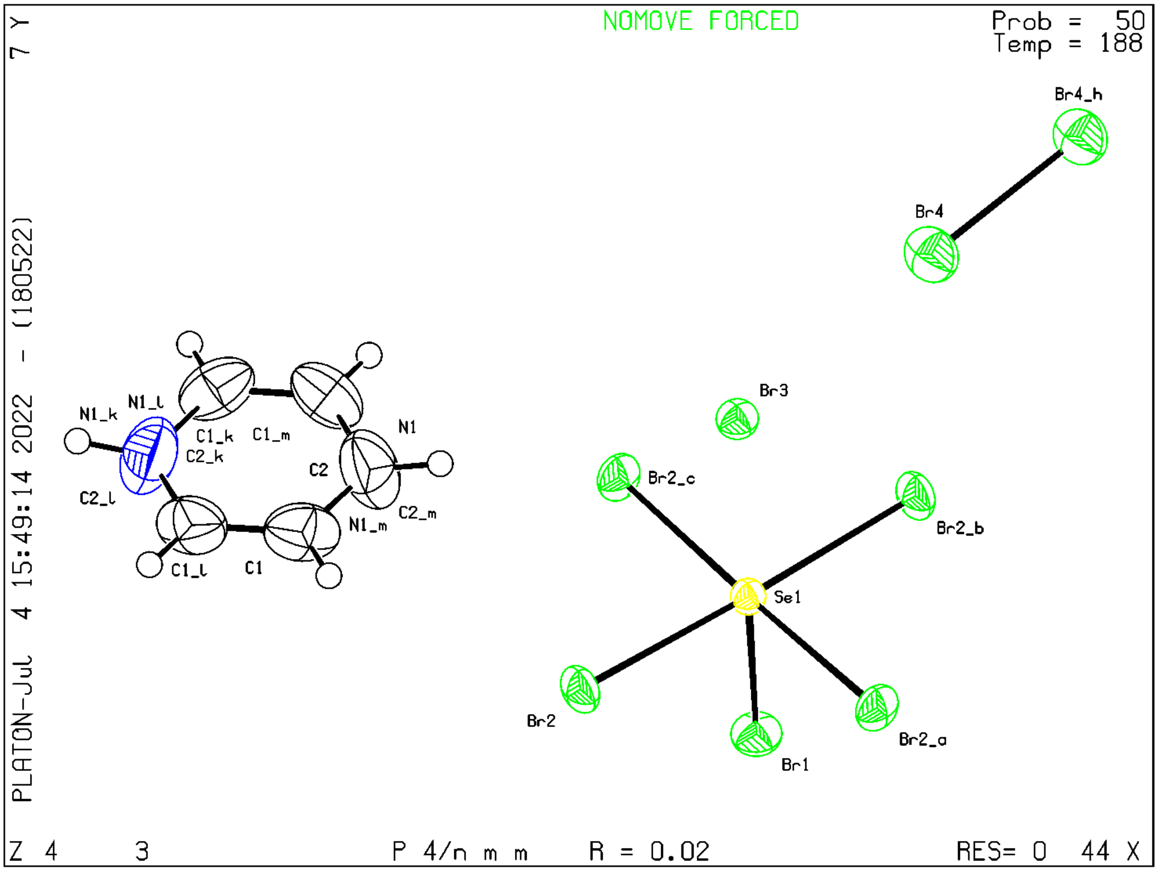

Supplement: Supplementary file 1 [file molecules-27-05355-s001.zip › checkcif 3.pdf]
