# Supplementary material for: Selenium(IV) Polybromide Complexes: Structural Diversity Driven by Halogen and Chalcogen Bonding
Source: Molecules. 2022 Aug 22;27(16):5355. doi: 10.3390/molecules27165355 (PMC9415170; doi:10.3390/molecules27165355)
Supplement: Supplementary file 1 [file molecules-27-05355-s001.zip › SI 04.07.docx]

**Selenium(IV) polybromide complexes: structural diversity driven by halogen and chalcogen bonding**

Nikita A. Korobeynikov, Andrey N. Usoltsev, Alexander S. Novikov, Pavel A. Abramov, Maxim N. Sokolov and Sergey A. Adonin*

**Table S1.** XRD experimental details.

|  | **(1)** | **(2)** | **(3)** | **(4)** |
| --- | --- | --- | --- | --- |
| Chemical formula | C_30_H_40_Br_21_N_5_Se_3_ | C_12_H_16_Br_8_N_2_Se | C_10_H_12_Br_10_N_2_Se | C_12_H_16_Br_8_N_2_Se |
| *M*_r_ | 2385.66 | 906.51 | 1038.28 | 906.51 |
| Crystal system, space group | Monoclinic, *C*2/*c* | Monoclinic, *C*2/*c* | Tetragonal, *P*4/*nmm* | Tetragonal, *P*4_1_2_1_2 |
| Temperature (K) | 150 | 150 | 188 | 150 |
| *a*, *b*, *c* (Å) | 11.0372 (9), 16.0823 (12), 33.061 (3) | 17.7961 (15), 7.4754 (6), 17.6525 (17) | 11.7570 (5), 11.7570 (5), 8.6464 (5) | 9.3744 (4), 9.3744 (4), 25.5866 (13) |
| α, β, γ (°) | 90, 94.534 (3), 90 | 90, 100.133 (5), 90 | 90, 90, 90 | 90, 90, 90 |
| *V* (Å^3^) | 5850.1 (8) | 2311.7 (4) | 1195.17 (12) | 2248.5 (2) |
| *Z* | 4 | 4 | 2 | 4 |
| µ (mm^-1^) | 16.26 | 15.45 | 18.29 | 15.89 |
| *T*_min_, *T*_max_ | 0.452, 0.746 | 0.540, 0.746 | 0.572, 0.746 | 0.577, 0.746 |
| No. of measured, independent and  observed [*I* > 2σ(*I*)] reflections | 44440, 6980, 5748 | 13577, 2561, 1919 | 16382, 1154, 955 | 42568, 2694, 2608 |
| *R*_int_ | 0.070 | 0.043 | 0.058 | 0.047 |
| θ values (°) | θ_max_ = 27.9, θ_min_ = 2.2 | θ_max_ = 27.1, θ_min_ = 2.3 | θ_max_ = 31.5, θ_min_ = 2.4 | θ_max_ = 27.9, θ_min_ = 2.3 |
| (sin θ/λ)_max_ (Å^-1^) | 0.658 | 0.642 | 0.735 | 0.659 |
| Range of *h*, *k*, *l* | -13 ≤ *h* ≤ 14  -21 ≤ *k* ≤ 21  -43 ≤ *l* ≤ 43 | -22 ≤ *h* ≤ 22  -9 ≤ *k* ≤ 9  -22 ≤ *l* ≤ 22 | -17 ≤ *h* ≤ 17  -17 ≤ *k* ≤ 17  -12 ≤ *l* ≤ 12 | -12 ≤ *h* ≤ 12  -12 ≤ *k* ≤ 12  -33 ≤ *l* ≤ 33 |
| *R*[*F*^2^ > 2σ(*F*^2^)], *wR*(*F*^2^), *S* | 0.063, 0.152, 1.31 | 0.046, 0.125, 1.09 | 0.024, 0.048, 1.09 | 0.013, 0.026, 1.05 |
| No. of reflections, parameters, restraints | 6980, 253, 12 | 2561, 124, 36 | 1154, 42, 0 | 2694, 106, 0 |
| H-atom treatment | H-atom parameters constrained | H-atom parameters constrained | Only H-atom coordinates refined | H-atom parameters constrained |
| Δρ_max_, Δρ_min_ (e Å^-3^) | 1.40, -1.81 | 1.20, -1.40 | 0.64, -0.88 | 0.37, -0.30 |
| Absolute structure | – | – | – | Flack x determined using 1011 quotients [(I+)-(I-)]/[(I+)+(I-)] (Acta Cryst. B69 (2013) 249-259). |
| Absolute structure parameter | – | – | – | -0.013 (8) |


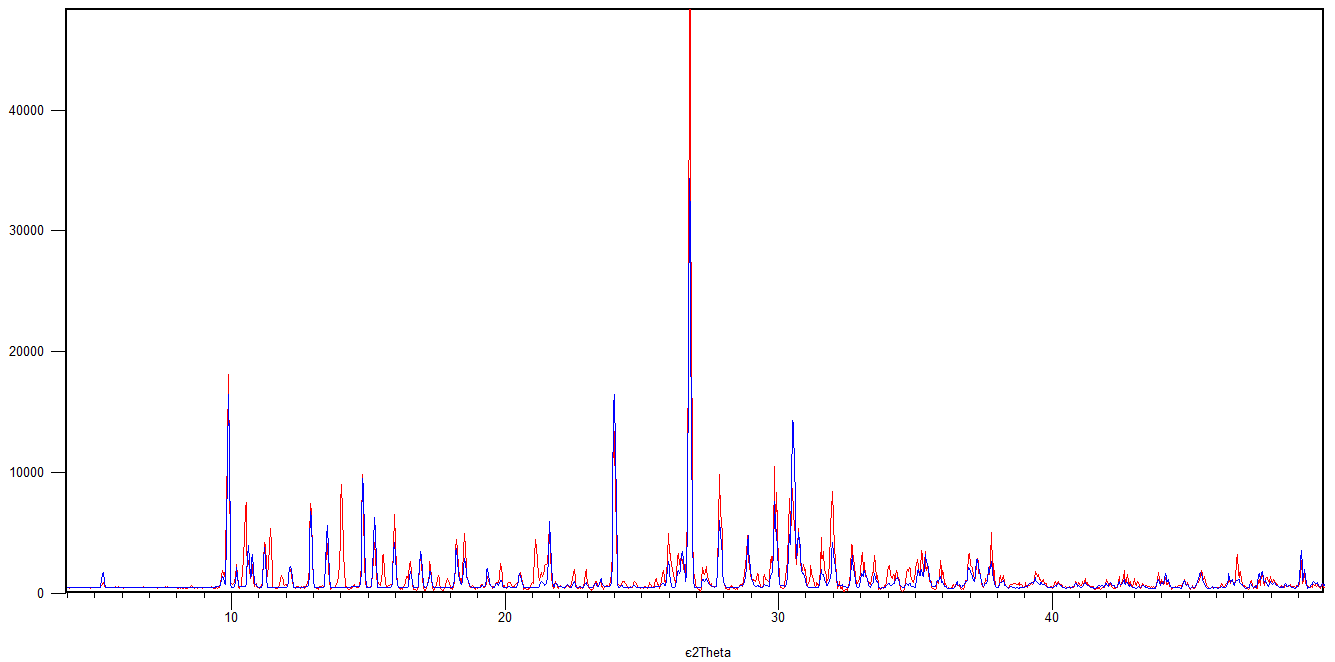


**Figure S1.** Comparison of experimental (red) and calculated (blue) PXRD patterns for 1.


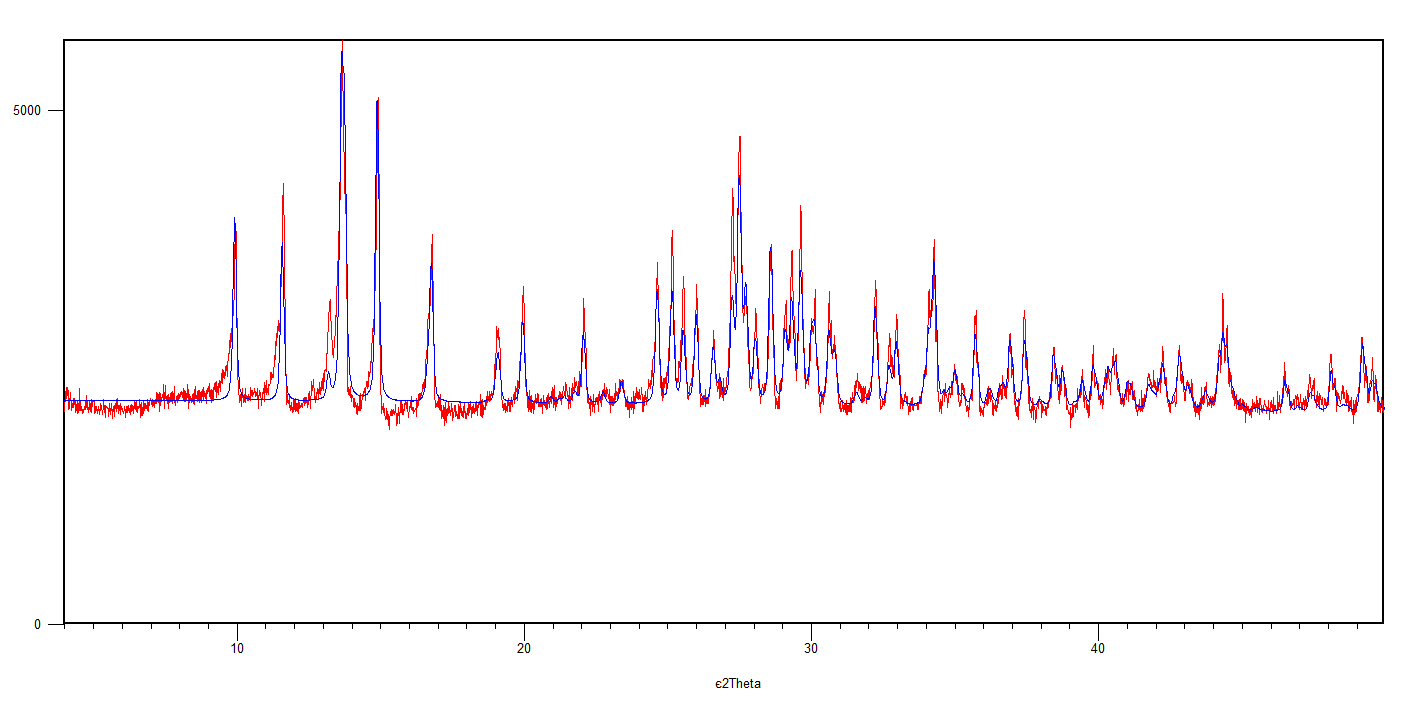


**Figure S2.** Comparison of experimental (red) and calculated (blue) PXRD patterns for 4.

Thermogravimetric analyses (TGA) were carried out on a TG 209 F1 Iris thermobalance (NETZSCH, Germany). The measurements were made in a helium flow in the temperature range of 30–350°C using the heating rate of 10°C min^-1^ the gas flow rate of 60 mL min^-1^ and open Al crucibles.


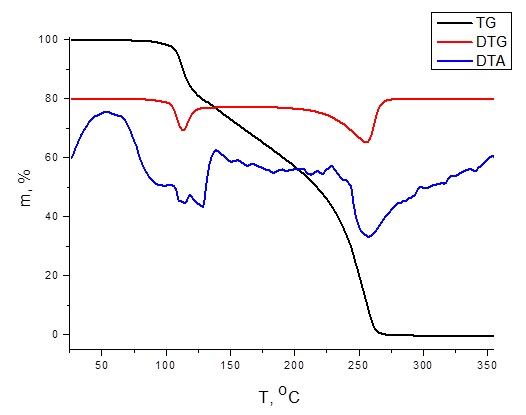


**Figure S3.** TG (black), DTG (red) and DTA (blue) curves for 4.

**Computational details**

The DFT calculations based on the experimental X-ray geometries have been carried out using the dispersion-corrected hybrid functional ωB97XD [Phys. Chem. Chem. Phys. 2008, 10, 6615.] with the help of Gaussian-09 [M. J. Frisch et al., in Gaussian 09, Revision C.01, Gaussian, Inc., Wallingford, CT, 2010.] program package. The Douglas–Kroll–Hess 2^nd^ order scalar relativistic calculations requested relativistic core Hamiltonian were carried out using the DZP-DKH basis sets [Mol. Phys. 2010, 108, 1965. || J. Chem. Phys. 2009, 130, 064108. || Chem. Phys. Lett. 2013, 582, 158. || J. Mol. Struct. - Theochem 2010, 961, 107.] for all atoms. The topological analysis of the electron density distribution has been performed by using the Multiwfn program (version 3.7) [J. Comput. Chem. 2012, 33, 580.]. The Cartesian atomic coordinates for model structures are presented in **Table S2.**

**Table S2**. Cartesian atomic coordinates for model supramolecular associates.

| Atom | X | Y | Z |
| --- | --- | --- | --- |
| **1** | | | |
| Se | 6.971153 | 12.061725 | 16.478769 |
| Br | 5.635317 | 13.246508 | 14.631829 |
| Br | 4.785998 | 11.187652 | 17.536706 |
| Br | 6.874380 | 14.165451 | 17.949664 |
| Br | 8.306989 | 10.876942 | 18.325710 |
| Br | 9.156308 | 12.935798 | 15.420832 |
| Br | 7.067926 | 9.957999 | 15.007874 |
| Br | 2.071835 | 9.731721 | 19.051105 |
| Br | -0.040773 | 8.916510 | 20.029285 |
| Br | -2.359122 | 8.093417 | 21.061845 |
| Br | 13.109035 | 9.731721 | 19.051105 |
| Br | 10.996427 | 8.916510 | 20.029285 |
| Br | 8.678078 | 8.093417 | 21.061845 |
| Br | 6.351871 | 6.350579 | 13.906434 |
| Br | 8.464479 | 7.165790 | 12.928254 |
| Br | 10.782827 | 7.988883 | 11.895694 |
| Se | 4.035700 | 5.674157 | 22.934163 |
| Br | 6.050880 | 6.668365 | 22.075289 |
| Br | 4.239529 | 3.653094 | 21.705506 |
| Br | 2.621911 | 6.786087 | 21.342313 |
| Br | 5.409361 | 4.572037 | 25.141988 |
| Br | 3.558479 | 7.854917 | 24.718154 |
| Se | 3.081259 | 5.674157 | 26.502146 |
| Br | 1.066079 | 6.668365 | 27.361019 |
| Br | 2.877430 | 3.653094 | 27.730803 |
| Br | 4.495048 | 6.786087 | 28.093995 |
| Br | 1.707597 | 4.572037 | 24.294320 |
| Br | 7.590435 | 1.690571 | 19.051105 |
| Br | 5.477827 | 0.875360 | 20.029285 |
| Br | 3.159478 | 0.052267 | 21.061845 |
| **3** | | | |
| Se | 8.817750 | 8.817750 | 4.462926 |
| Br | 8.817750 | 8.817750 | 2.102891 |
| Br | 7.009053 | 7.009053 | 4.533135 |
| Br | 10.626447 | 10.626447 | 4.533135 |
| Br | 10.626447 | 7.009053 | 4.533135 |
| Br | 7.009053 | 10.626447 | 4.533135 |
| Br | 8.817750 | 8.817750 | 7.511560 |
| Br | 10.944121 | 10.944121 | 8.383204 |
| Br | 12.569879 | 12.569879 | 8.909596 |
| Se | 14.696250 | 14.696250 | 4.183474 |
| Br | 14.696250 | 14.696250 | 6.543509 |
| Br | 16.504947 | 12.887553 | 4.113265 |
| Br | 12.887553 | 16.504947 | 4.113265 |
| Br | 12.887553 | 12.887553 | 4.113265 |
| Br | 16.504947 | 16.504947 | 4.113265 |
| Se | 8.817750 | 8.817750 | 13.109326 |
| Br | 8.817750 | 8.817750 | 10.749291 |
| Br | 7.009053 | 7.009053 | 13.179535 |
| Br | 10.626447 | 10.626447 | 13.179535 |
| Br | 10.626447 | 7.009053 | 13.179535 |
| Br | 7.009053 | 10.626447 | 13.179535 |
| **4** | | | |
| Se | 6.023146 | 3.351254 | 19.189950 |
| Br | 5.414466 | 2.795634 | 16.754362 |
| Br | 4.177420 | 5.196980 | 19.189950 |
| Br | 7.745785 | 5.054208 | 18.286743 |
| Br | 7.800157 | 1.574243 | 19.189950 |
| Br | 6.578766 | 3.959934 | 21.625538 |
| Br | 4.320192 | 1.628615 | 20.093157 |
| Br | 10.074386 | 7.068673 | 18.918220 |
| Br | 11.680127 | 8.674414 | 19.461680 |

**
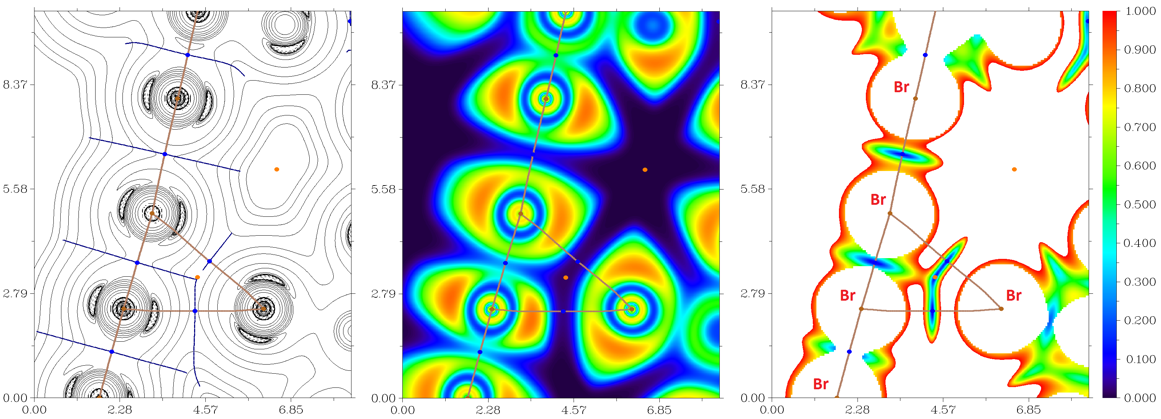
**

**Figure S4**. Contour line diagram of the Laplacian of electron density distribution ∇^2^ρ(**r**), bond paths, and selected zero-flux surfaces (left panel), visualization of electron localization function (ELF, center panel) and reduced density gradient (RDG, right panel) analyses for noncovalent interactions Br···Br in the X-ray structure **1**. Bond critical points (3, –1) are shown in blue, nuclear critical points (3, –3) – in pale brown, ring critical points (3, +1) – in orange, bond paths are shown as pale brown lines, length units – Å, and the color scale for the ELF and RDG maps is presented in a.u.


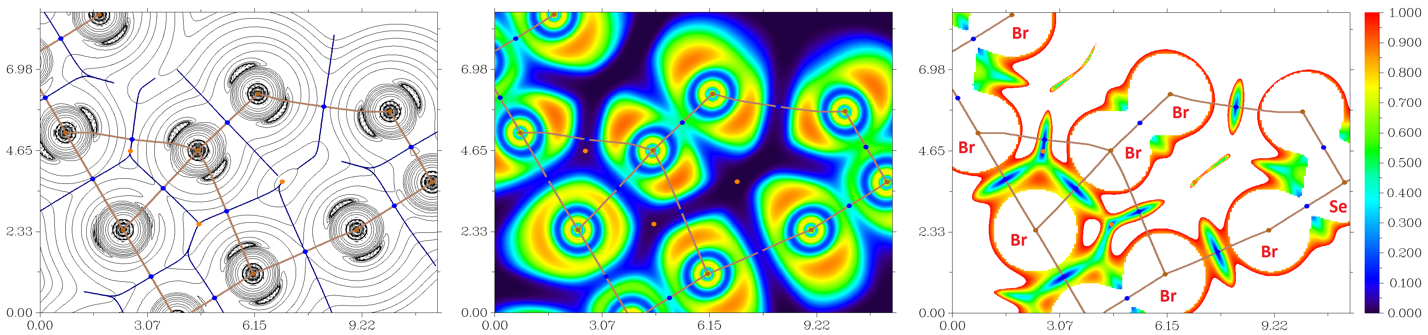


**Figure S5**. Contour line diagram of the Laplacian of electron density distribution ∇^2^ρ(**r**), bond paths, and selected zero-flux surfaces (left panel), visualization of electron localization function (ELF, center panel) and reduced density gradient (RDG, right panel) analyses for noncovalent interactions Br···Br and Se···Br in the X-ray structure **3**. Bond critical points (3, –1) are shown in blue, nuclear critical points (3, –3) – in pale brown, ring critical points (3, +1) – in orange, bond paths are shown as pale brown lines, length units – Å, and the color scale for the ELF and RDG maps is presented in a.u.


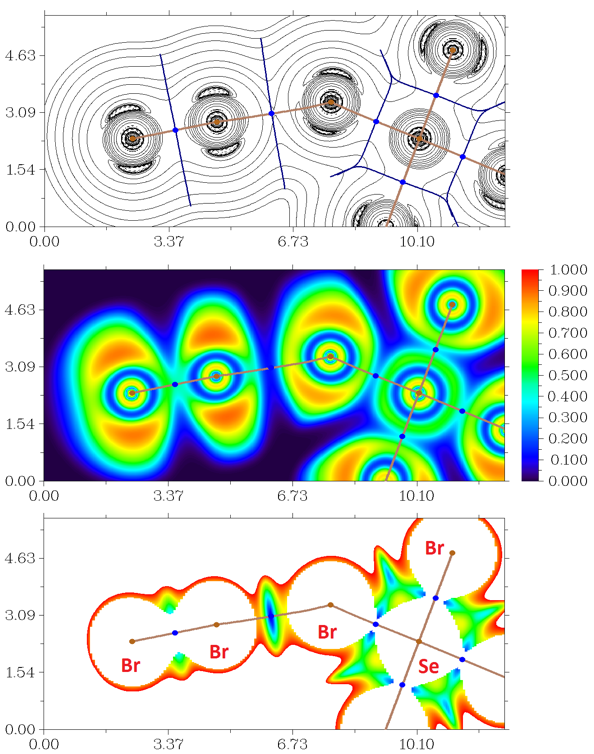


**Figure S6**. Contour line diagram of the Laplacian of electron density distribution ∇^2^ρ(**r**), bond paths, and selected zero-flux surfaces (top panel), visualization of electron localization function (ELF, center panel) and reduced density gradient (RDG, bottom panel) analyses for noncovalent interactions Br···Br in the X-ray structure **4**. Bond critical points (3, –1) are shown in blue, nuclear critical points (3, –3) – in pale brown, bond paths are shown as pale brown lines, length units – Å, and the color scale for the ELF and RDG maps is presented in a.u.
